# Ultrasound grayscale ratio: a reliable parameter for differentiating between papillary thyroid microcarcinoma and micronodular goiter

**DOI:** 10.1186/s12902-022-00994-9

**Published:** 2022-03-24

**Authors:** Yun Gong, Xiuzhen Yao, Lifang Yu, Peiying Wei, Zhijiang Han, Jianhua Fang, Weiqun Ao, Chenke Xu

**Affiliations:** 1 Department of Pediatrics, Affiliated Hangzhou First People’s Hospital, Zhejiang University School of Medicine, Zhejiang, Hangzhou China; 2 Department of Ultrasound, Shanghai Putuo District People’s Hospital, Shanghai, China; 3 Department of Ultrasound, Affiliated Hangzhou First People’s Hospital, Zhejiang University School of Medicine, No. 261, Huansha Road, Shangcheng District, Zhejiang, 310006 Hangzhou China; 4 Department of Radiology, Affiliated Hangzhou First People’s Hospital, Zhejiang University School of Medicine, Hangzhou Zhejiang, China; 5 Department of Radiology, Tongde Hospital of Zhejiang Province, No.234, Gucui Road, Zhejiang, 310012 Hangzhou China

**Keywords:** Ultrasound grayscale ratio, Papillary thyroid microcarcinoma, Micronodular goiter

## Abstract

**Background:**

The present study aimed to quantify and differentiate the echo levels of papillary thyroid microcarcinomas (PTMCs) and micronodular goiters (MNGs) using the ultrasound grayscale ratio (UGSR) and to investigate the repeatability of UGSR.

**Methods:**

The ultrasound (US) data of 241 patients with 265 PTMCs and 141 patients with 168 MNGs confirmed by surgery and pathology were retrospectively analyzed. All patients had received outpatient ultrasonic examination and preoperative ultrasonic positioning. The RADinfo radiograph reading system was used to measure the grayscales of PTMC, MNG, and thyroid tissues at the same gain level, and the UGSR values of the PTMC, MNG, and thyroid tissue were calculated. The patients were divided into outpatient examination, preoperative positioning, and mean value groups, and the receiver operating characteristic (ROC) curves were calculated to obtain the optimal UGSR threshold to distinguish PTMC from MNG. The interclass correlation coefficient (ICC) was used to assess the consistency of UGSR measured in three groups.

**Results:**

The UGSR values of the PTMC and MNG were 0.56 ± 0.14 and 0.80 ± 0.19 (*t* = 5.84, *P* < 0.001) in the outpatient examination group, 0.55 ± 0.14 and 0.80 ± 0.19 (*t* = 18.74, *P* < 0.001) in the preoperative positioning group, and 0.56 ± 0.12 and 0.80 ± 0.18 (t = 16.49, *P* < 0.001) in the mean value group. The areas under the ROC curves in the three groups were 0.860, 0.856, and 0.875, respectively. When the UGSR values for the outpatient examination, preoperative positioning, and mean value groups were 0.649, 0.646, and 0.657, respectively, each group obtained its largest Youden index. A reliable UGSR value was obtained between the outpatient examination and preoperative positioning groups (ICC = 0.79, *P* = 0.68).

**Conclusion:**

UGSR is a simple and repeatable method to distinguish PTMC from MNG, and hence, can be widely applicable.

## Background

Thyroid lesions are divided into five categories based on the comparison of echogenicity between the cervical strap muscle and thyroid: anechoic, markedly hypoechoic (echogenicity lower than that of the cervical strap muscle), hypoechoic (echogenicity between that of the cervical strap muscle and thyroid parenchyma), isoechoic (with the same echogenicity as the thyroid parenchyma), and hyperechoic (more echoic than the thyroid parenchyma) [1–3]. The diagnostic values of the hypoechoic and markedly hypoechoic categories have been widely accepted for thyroid malignant nodules [1, 4–7]. However, distinguishing between hypoechoic and markedly hypoechoic categories is a subjective process that is influenced by the observer’s judgment. Thus, their diagnostic values for malignant nodules vary across studies: the sensitivity and specificity are 79–93.8% and 21.8–61% for the hypoechoic category, respectively, and 17.1–41.4% and 90.9–98.8% for the markedly hypoechoic category, respectively [8–11]. Accumulating evidence suggested that the specificity of the hypoechoic category and the sensitivity of the markedly hypoechoic category are insufficient for clinical diagnosis. The quantification of the echogenicity of nodules eliminates the differences due to subjective judgment. Additionally, as the computer intelligent auxiliary diagnosis progresses, the quantification of grayscale values contributes to rapid and accurate diagnosis on a large scale.

The absolute ultrasound grayscale value is affected by the scanning apparatus, scan gain, dynamic range, frequency, and operators [12]. Thus, unlike computed tomography (CT) scans, the absolute ultrasound grayscale value is not suitable for the diagnosis of thyroid lesions. However, based on standardized ultrasound (US) scanning, even if the aforementioned influence factors are changed, the grayscale value of nodules and the surrounding thyroid tissues at the same gain level are consistent with a stable correlation. Therefore, it is impossible to assess the echogenicity using the absolute grayscale value. The ultrasound grayscale ratio (UGSR) is the grayscale ratio of pathological tissues to the surrounding normal tissues at the same gain level. Moreover, whether the UGSR is repeatable in different US examinations under various operating conditions is not yet been reported. In the present study, we compared the UGSRs in the outpatient US group, preoperative positioning US group, and the mean value group among the same patient population to explore the repeatability of UGSR and its diagnostic efficacy in differentiating between PTMCs and MNGs. This could provide a basis for further diagnosis and clinical treatment.

## Methods

### Subjects

**Fig. 1:**
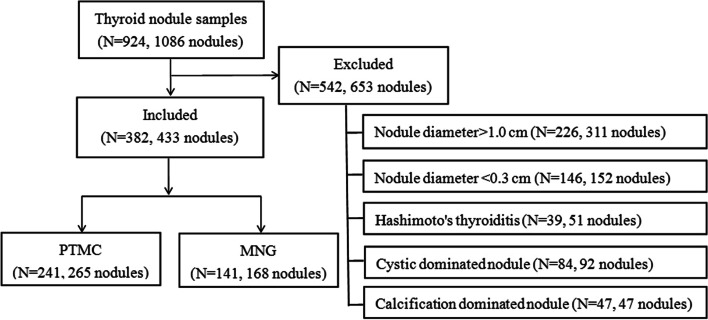
Schematic of the study population

This study was approved by the ethics committee of Affiliated Hangzhou First People’s Hospital, Zhejiang University School of Medicine. All patients or their legally authorized representatives provided written informed consent prior to participation in the study. All the procedures were performed in accordance with the Declaration of Helsinki and relevant policies in China. A total of 924 pathologically-confirmed cases of patients with 1086 thyroid nodules were recruited between January 2017 and October 2020. All patients were examined by outpatient and preoperative positioning US. The interval between the two examinations was ≤15 days. We excluded the thyroid nodules with a diameter > 1.0 cm because their internal echogenicity could be uneven. Nodules with a diameter < 0.3 cm were also excluded to avoid the effect of the partial volume effect on the measurement. Also, cystic-dominated nodules (the cystic component accounted for > 50% of the nodule), calcification-dominated nodules (the soft tissues of nodules could not be measured due to obvious calcification), and nodules complicated with Hashimoto’s thyroiditis were excluded from the study [13–16]. Finally, the cohort comprised 382 patients with 433 nodules, including 84 men and 298 women, with an average age of 48 ± 11 (range: 23–78) years (Fig. 1).

### US examination

The thyroid lesions were examined by US on one of the following ultrasonic diagnostic scanners: MyLab 70 XVG (Genoa, Italy), Esaote MyLab Classic C (Genoa, Italy), Esaote Mylab 90 (Genoa, Italy), GE Voluson E8 (Tiefenbach, Austria), GE Voluson E10 (Tiefenbach, Austria), GE logic E9 (Wauwatosa, USA), Philips HD 11 XE (Amsterdam, The Netherlands), Philips HDI 5000 (Amsterdam, The Netherlands), Siemens S2000 (Buffalo Grove, USA), and Mindray Resona7 (Shenzhen, China). Then, 5–13 MHz broadband linear array probes were used for this study, and the central frequency was 7.5 MHz. The patients were placed in a supine position, exposing the anterior cervical region, following which the transverse, longitudinal, and oblique scans were performed.

### Image analysis

**Fig. 2:**
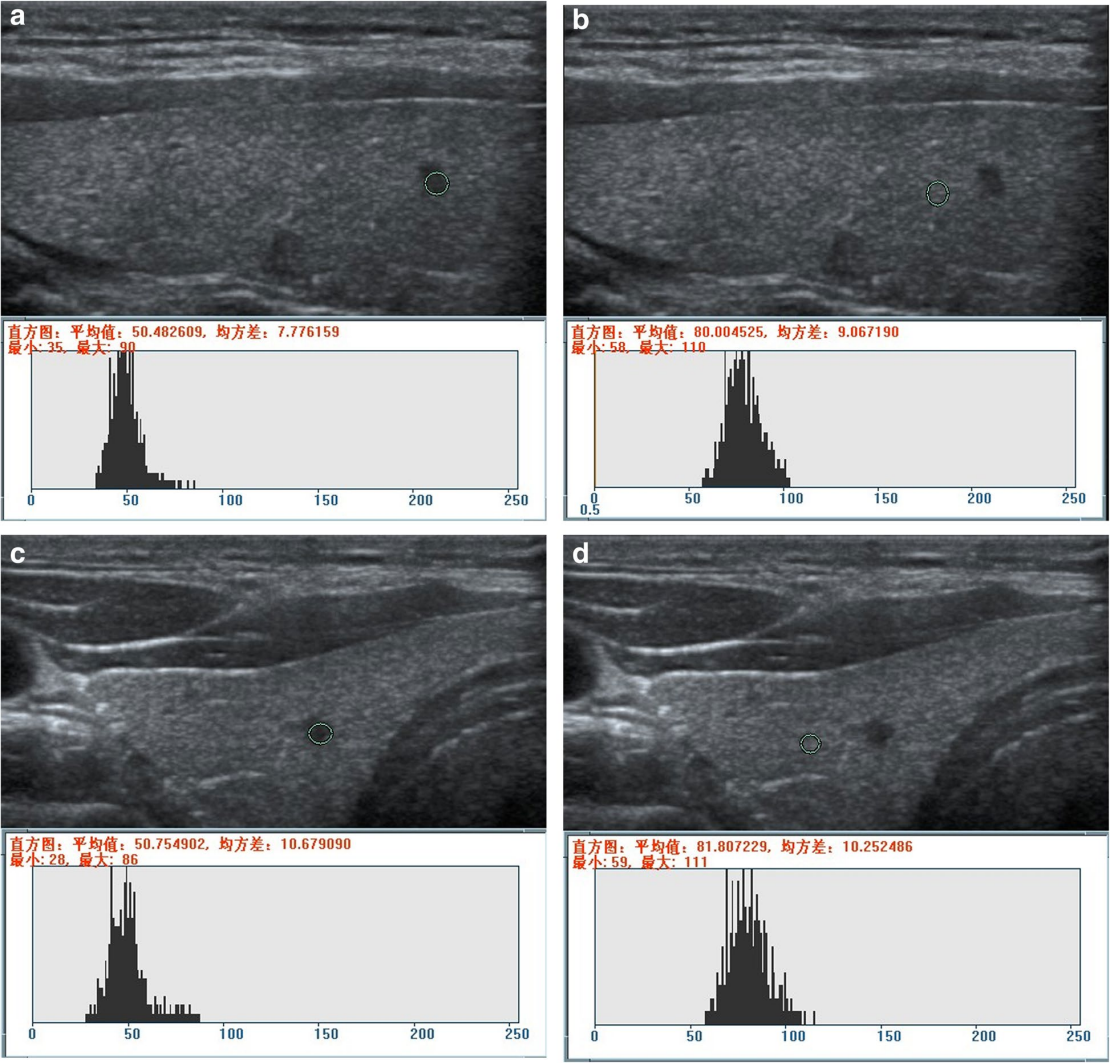
Measurement of the UGSR value for PTMC on the right lobe of the thyroid. **a** and **b** (ultrasonic apparatus: Philips HD 11 XE): In the outpatient examination group, a longitudinal scan revealed grayscales of 50.482609 and 80.04525 for PTMC and thyroid tissues at the same gain level, respectively. The UGSR value was 0.630997 (50.482609/80.04525). **c** and **d** (ultrasonic apparatus: Siemens S2000): In the preoperative positioning group, the transverse scan showed grayscales of 50.754902 and 81.807229 for PTMC and thyroid tissues at the same gain level, respectively. The UGSR value was 0.620421 (50.754902/81.807229), and the interval was five days. The difference in the UGSR was 0.010576 (0.630997–0.620421) between the two groups (the outpatient examination and preoperative positioning groups)

**Fig. 3:**
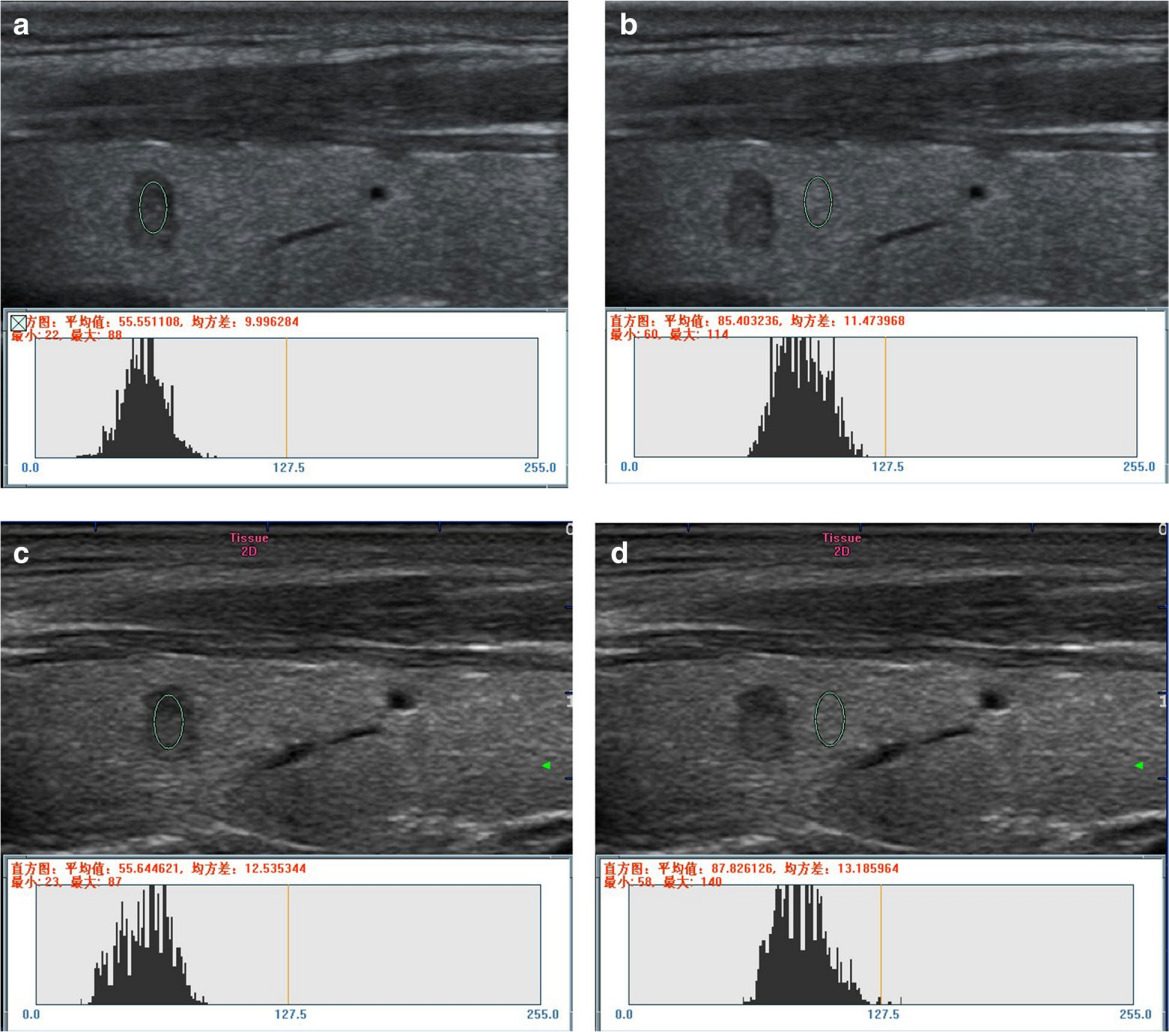
Measurement of the UGSR value for PTMC on the right lobe of the thyroid. **a** and **b** (ultrasonic apparatus: Mindray Resona7): In the outpatient examination group, a longitudinal scan revealed grayscales of 55.551108 and 85.403236 for PTMC and thyroid tissues at the same gain level, respectively. The UGSR value was 0.650457 (55.551108/85.403236). **c** and **d** (ultrasonic apparatus: MyLab Classic C): In the preoperative positioning group, a longitudinal scan showed grayscales of 55.644621 and 87.826126 for PTMC and thyroid tissues at the same gain level, respectively. The UGSR value was 0.633577 (55.644621/87.826126), and the interval was seven days. The difference of the UGSR was 0.016880 (0.650457–0.633577) between the two groups (the outpatient examination and the preoperative positioning groups)

**Fig. 4:**
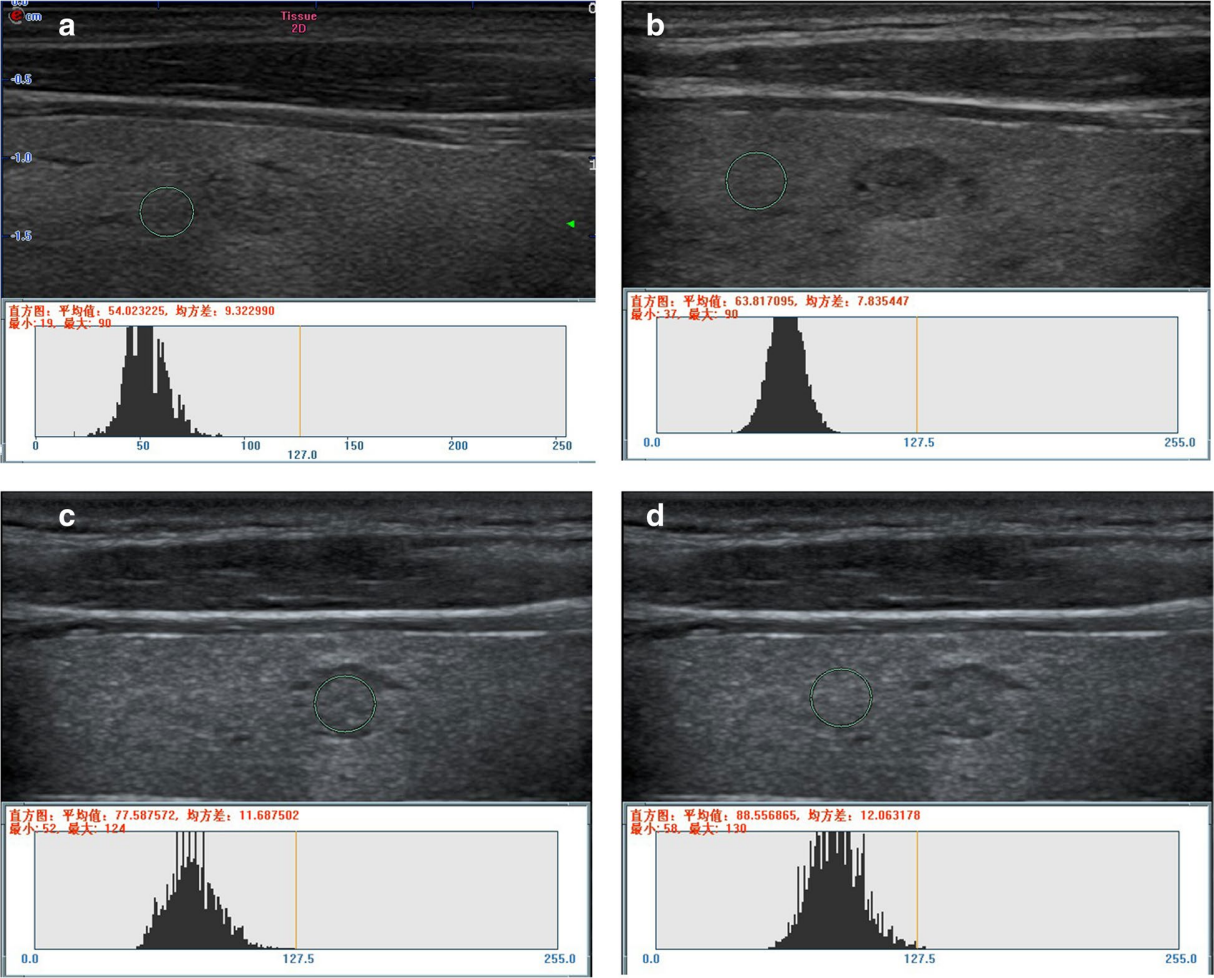
Measurement of the UGSR value for MNG on the left lobe of the thyroid. **a** and **b** (ultrasonic apparatus: Mindray Resona7): In the outpatient examination group, a longitudinal scan revealed grayscales of 54.667273 and 63.817095 for MNG and thyroid tissues at the same gain level, respectively. The UGSR value was 0.856624 (54.667273/63.817095). **c** and **d** (ultrasonic apparatus: Esaote Mylab 90): In the preoperative positioning group, a longitudinal scan shows grayscales of 77.587572 and 88.556865 for MNG and thyroid tissues at the same gain level, respectively. The UGSR value was 0.876133 (77.587572/88.556865), and the interval was 10 days. The difference of the UGSR was 0.019509 (0.876133–0.856624) between the two groups (the outpatient examination and preoperative positioning groups)

The ultrasonographic data, selected from the Picture Archiving and Communication Systems database, were analyzed by two radiologists with 14 and 16 years of experience, respectively. The US grayscale values of the PTMC, MNG, and thyroid tissues at the same gain level were retrospectively reviewed using the RADinfo radiograph reading system (Yilaida, Zhejiang Province, China), and the corresponding UGSR values were calculated. Subsequently, the patients were divided into the outpatient examination group, preoperative positioning group, and the mean value group (the mean value of the first two groups). The optimal UGSR value for differentiating PTMC from MNG was determined by analyzing the receiver operating characteristic (ROC) curves. The calcification and necrosis regions were avoided while measuring the regions of interest (ROI). For nodules with homogeneous echo, an ROI > 1/2 of the largest areas of nodules was selected, for nodules with heterogeneous echo, an ROI > 1/2 of echo-dominated areas within the regions was selected, and for measuring the grayscales of surrounding normal thyroids, the ROIs of nodules at the same gain level and size were selected (Figs. 2, 3 and 4).

### Statistical analysis

SPSS for Windows version 22.0 software package (IBM Co., Armonk, NY) was used to generate the ROC curves for differentiating PTMC from MNG. The UGSR threshold was determined by measuring the area under the ROC curve (AUC). The differences in the UGSR value between and within groups were compared using either the analysis of variance or Student’s t-test. The reliability of the UGSR was analyzed in the outpatient examination and preoperative positioning groups. *P*-values < 0.05 were considered to be statistically significant.

## Results

### UGSRs between PTMCs and MNGs in the three groups

The 433 nodules comprised 265 PTMCs from 241 patients and 168 MNGs from 141 patients. Among them, 33 cases presented both PTMC and MNG. As shown in Table 1, the UGSR values of the PTMC and MNG were 0.56 ± 0.14 and 0.80 ± 0.19 (*t* = 5.84, *P* < 0.001) in the outpatient examination group, 0.55 ± 0.14 and 0.80 ± 0.19 (*t* = 18.74, *P* < 0.001) in the preoperative positioning group, and 0.56 ± 0.12 and 0.80 ± 0.18 (*t* = 16.49, *P* < 0.001) in the mean value group, respectively.

**Table 1:** Comparisons of UGSR for distinguishing PTMC from MNG in different groups

Group	Number		UGSR		t	*P*
	PTMC	MNG	PTMC	MNG		
Outpatient examination	265	168	0.56±0.14	0.80±0.19	5.84	<0.001
Preoperative positioning	265	168	0.55±0.14	0.80±0.19	18.74	<0.001
Mean value	265	168	0.56±0.12	0.80±0.18	16.49	<0.001

PTMC and MNG data are presented as the mean ± standard deviation

### Diagnostic efficacies of UGSRs in the three groups

**Fig. 5:**
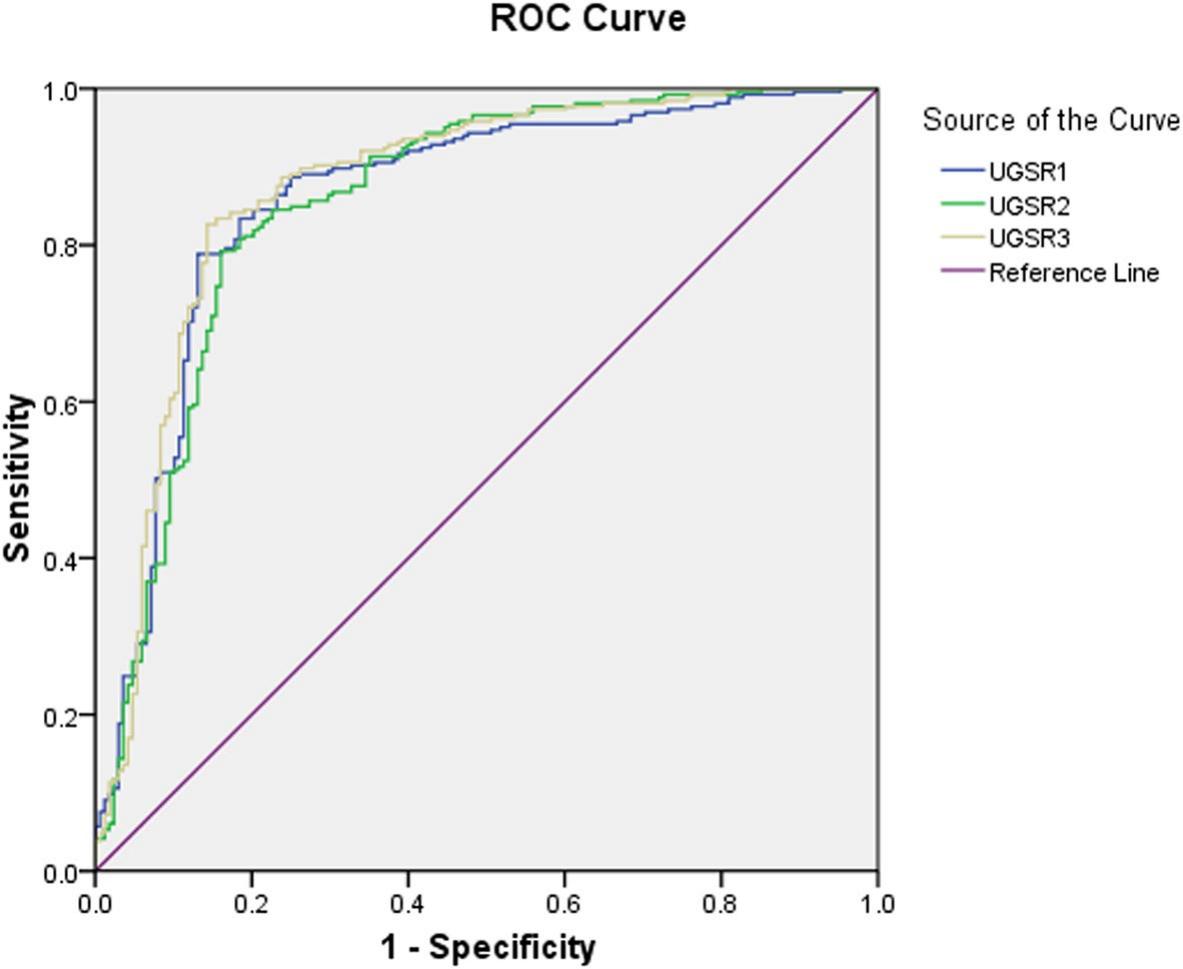
ROC curve of the UGSR value to distinguish between PTMC and MNG (UGSR1: outpatient examination group, UGSR2: preoperative positioning group, UGSR3: mean value group)

The AUC for distinguishing PTMC and MNG was 0.860 in the outpatient examination group, 0.856 in the preoperative positioning group, and 0.875 in the mean value group. When the UGSR for the outpatient examination, preoperative positioning, and mean value groups was 0.649, 0.646, and 0.657, respectively, each group exhibited the largest Youden index (0.658, 0.631, and 0.683, respectively). The sensitivity and specificity for diagnosing PTMC in the outpatient examination, preoperative positioning, and the mean value groups were 78.9 and 86.9%, 79.2 and 83.9%, and 82.6 and 85.7%, respectively (Fig. 5).

### Consistency analysis

A reliable UGSR value was obtained between the outpatient examination and preoperative positioning groups (ICC = 0.79, *P* = 0.68).

## Discussion

Herein, we compared the data of two ultrasound examinations (outpatient US and preoperative positioning US) in the same patient population and analyzed the repeatability of UGSR in different scanners with respect to gain levels, dynamic range, frequency, and operators. We also discussed its diagnostic efficacy for differentiating between PTMC and MNG. The results showed that UGSR in the two ultrasound examinations had good consistency. The AUC, sensitivity, and specificity of UGSRs for diagnosing PTMCs were similar, and the diagnostic efficiency was significantly higher than that reported previously [14–16]. A few studies reported the quantification of US echogenicity intensity. In 2013, Erol et al. [17] proposed the concept of lesion echogenicity ratio (LER): the ratio of echogenicity values of the neighboring fat lobule to the echogenicity values of lesions. The study demonstrated that the LER was significantly higher in malignant lesions than in benign lesions. Although this study quantified the echogenicity of mammary glands, the following two deficiencies were noted: 1) Physiological factors, such as menstrual cycle and age, affect the echogenicity of the breast; 2) The neighboring fat lobules were scattered, and the results estimated were variable in different regions. Compared to the breast tissue, the echogenicity of the thyroid may be barely affected by the above factors. The echogenicity of normal thyroid tissue is uniform, and that of benign and malignant lesions is different, which is rather suitable for quantitative analysis.

In 2018, we used UGSR to identify PTMCs and MNGs, and the results showed that the AUC, best cutoff value, sensitivity, and specificity were 0.895, 0.72, 87.0, and 80.4%, respectively [18]. In 2019, Chen et al. used the UGSR to distinguish between papillary thyroid carcinomas and nodular goiter with different sizes examined by the same US scanner. The results showed that the AUC for diagnosing small-sized PTCs (0.3–1 cm) was 0.919, and the corresponding sensitivity and specificity were 97.5 and 72.4%, respectively [19]. The findings also supported our previous study and demonstrated that the application of UGSR in different medical centers was repeatable. In addition, the repeatability in the same medical center is a critical indicator to measure whether UGSR can be promoted and applied widely. In this study, the results of the two ultrasound examinations were compared in the same patient population. The results showed that the diagnostic efficacy for the three groups was better than those for previously reported data [14, 20, 21]. Thus UGSR had good stability and diagnostic efficacy in different and same centers. It could quantify the echo level and reduce the difference in the subjective judgment of observers, which could be used as a major parameter for diagnosing PTMCs.

The present study has several limitations. First, the internal echogenicity of nodules with a diameter > 1 cm is uneven, which might affect the measurement; hence, these nodules were not included in this study. We will further expand the sample size for a comparative study on nodules of different sizes. Second, there may be some differences in ROI selection and measurement for a small number of nodules with heterogeneous echogenicity or normal thyroid tissues with heterogeneous echogenicity due to technical issues. In this study, the ROI was selected and measured by two senior imaging specialists, which greatly reduced this deviation. Finally, the selection bias was inevitable because this was a retrospective study, and the data were obtained from a single medical institute. Therefore, a prospective study in multiple medical institutes is necessary to validate our findings.

## Conclusion

In conclusion, UGSR maintains stability under the influence of various factors, such as different apparatus, gain levels, dynamic ranges, frequencies, and operators. It is a reliable parameter to distinguish PTMC from MNG that is suitable for popularization and application.

## Data Availability

All data generated or analyzed during this study are included in this article.

## Abbreviations

ICC: Intraclass correlation coefficient
LER: Lesion echogenicity ratio
MNG: Micronodular goiters
PTMC: Papillary thyroid microcarcinomas
ROC: Receiver operating characteristic
ROI: Region of interest
UGSR: Ultrasound grayscale ratio
US: Ultrasound
